# Role of Creativity in the Effectiveness of Cognitive Reappraisal

**DOI:** 10.3389/fpsyg.2017.01598

**Published:** 2017-09-15

**Authors:** Xiaofei Wu, Tingting Guo, Tengteng Tang, Baoguo Shi, Jing Luo

**Affiliations:** ^1^Beijing Key Laboratory of Learning and Cognition, The Collaborative Innovation Center for Capital Education Development, Department of Psychology, Capital Normal University Beijing, China; ^2^Key Laboratory of Mental Health, Institute of Psychology, Chinese Academy of Sciences Beijing, China

**Keywords:** cognitive reappraisal, creativity, negative emotions, regulation, effectiveness

## Abstract

As a well-recognized and widely adopted emotional regulation strategy, cognitive reappraisal has generally been proven to be efficient. However, the cognitive mechanism underlying regulatory efficiency, particularly the role of creativity, in cognitive reappraisal is unclear. Although previous studies have evaluated the relationship between creativity and reappraisal from the perspectives of generation (i.e., generating cognitive reappraisals and generating creative ideas involve similar cognitive neural networks) and individual differences (i.e., the ability to generate different cognitive reappraisals can be predicted by scores on creativity-related tests), how cognitive reappraisal’s efficiency can be related to creativity is still unknown. In this research, we assessed the relationship between cognitive reappraisal’s creativity and its effectiveness in regulating negative emotion. In Study 1, participants were asked to generate reappraisals of negative stimuli and then evaluate the creativity and regulatory effectiveness of these reappraisals. The results indicated positive correlation between creativity rating and regulatory effectiveness, but we found that it was difficult for the participants to generate highly creative reappraisals on their own. Therefore, in Study 2, we showed participants well-prepared reappraisal materials that varied in their creativity and asked them to evaluate their regulatory effectiveness and creativity. The results suggested that creativity and appropriateness were significant predictors of the regulating effects of the reappraisal and that creativity was the most dominant predictor. In summary, both experiments found a positive correlation between reappraisal’s creativity and effectiveness, thus implying that creativity plays an important role in reappraisal.

## Introduction

The ability to control effective response and to initiate more adaptive behavior has important consequences for our physical and mental well-being (Gross and John, 2003). Cognitive reappraisal, a linguistic strategy that alters the trajectory of emotional responses by reformulating the meaning of a situation (Gross, 1998), has been regarded as an effective method for regulating negative emotion (Aldao et al., 2010; Webb et al., 2012). However, the cognitive mechanisms mediating the generation of cognitive reappraisal and its effectiveness in regulating emotion remain to be specified. A recent meta-analysis of neuroimaging studies of cognitive reappraisal found that prefrontal and parietal control regions exert their effects through changes in the lateral temporal areas associated with semantic and perceptual representations (Buhle et al., 2014), thus supporting a mechanism of representational change for cognitive reappraisal to regulate emotion (Ochsner and Gross, 2005, 2007; Bebko et al., 2011). In other words, the process of cognitive reappraisal, by its very nature, means the process of changing or altering the mental set or the information-processing bias one uses to represent the situation. As an abstract representation of the encountered problem that problem solvers retain in their mind, the problem space could be classified as well-defined or ill-defined, according to the constraints that are formulated for the solution (Simon and Newell, 1971; Simon, 1973; Jaarsveld and Leeuwen, 2005; Jaarsveld et al., 2010, 2012, 2015; Jaarsveld and Lachmann, 2017). Accordingly, a change in the mental representation of a problem space can occur in two different ways (Simon and Newell, 1971). In the well-defined problem situation, in which the rules and mental operators are clear and limited, a change in strategy is explicit and directly available. For example, in the Wisconsin Card Sorting Test (WCST), a change in sorting strategies is always made among several limited selections (e.g., color, number, or shape), and this type of change is found to be mainly mediated by the neural network for cognitive control and executive function, particularly the prefrontal cortex (PFC) (e.g., Monchi et al., 2001). Another type of mental set or representation change, however, is made under an ill-defined problem situation, such as the one encountered in divergent thinking, in which the rules and mental operators are highly uncertain and the stepwise application of mental operators based on ordinary reasoning (e.g., to derive the possible applications of an empty can based on its major function) typically cannot result in truly original solutions. Unlike the ordinary representational change in the well-defined problem situation, which largely depends on the executive function, the representational change in the ill-defined problem situation typically occurs in a creative or insightful manner. More, in open or ill-defined problem space, cognitive productions tend to be more original, hence, more creative, than in well-defined or closed problem space. Therefore, changes in mental representation made under conditions of an ill-defined problem space, can result in more original solutions than changes made in a well-defined space (Jaarsveld and Lachmann, 2017). The ill-defined problem has been found to be facilitated to a certain extent by inhibition or dysfunction in the executive neural network (Reverberi et al., 2005; Radel et al., 2015) as well as by the participation of the default mode network (DMN) and medial temporal lobe (MTL) association and memory systems (Takeuchi et al., 2012; Duff et al., 2013). Regarding cognitive reappraisal, studies have indicated that the tactics of reappraisal can be categorized into limited types, such as change current circumstances, reality challenge, change future consequences, and distancing (McRae et al., 2012), thus implying the possibility of ordinary change in reappraisal. However, it is far more plausible that the change will be a creative or insightful one, given that it essentially requires an individual to search for and find new perspectives, which are not typically implicated by the problem situation, and to adopt new strategies, which can be highly task-specific for the new problem at hand (e.g., thinking through a new reappraisal of a new affective picture).

More importantly, recent studies have found connections between cognitive reappraisal and creativity from the perspective of individual differences (Weber et al., 2014) and generation processes (Fink et al., 2016). While adopting concepts from the studies on creative divergent thinking, Weber et al. (2014) developed the Reappraisal Inventiveness Test (RIT) to investigate individual differences in the ability to generate different reappraisals for an anger-eliciting situation, finding that the RIT can be positively associated with openness to experience and tests that measure divergent thinking. Fink et al. (2016) find that the generation of cognitive reappraisal is associated with a brain activation pattern (the pattern of alpha power) similar to that observed in verbal creative ideation but with higher cognitive control and fewer spontaneous imaginative thought processes relative to the latter; this finding thus implies that the representational change in cognitive reappraisal, in terms of generation processes, can also be creative. However, as an emotional regulation strategy, the goal of an individual in generating cognitive reappraisal is to change his/her emotions, particularly negative emotions. Therefore, in addition to the perspective of individual differences and generation processes, the effect of emotional regulation should also be considered. Only if we find a correlation between reappraisal’s creativity and its effectiveness in regulating emotion can we conclude that the representational change in cognitive reappraisal is creative. The roles of insight and creativity in psychological therapy have been widely recognized by therapists of various theoretical orientations (Hill, 2007; Novotney, 2013), particularly those engaged in cognitive behavioral therapy (CBT) (Elliott et al., 1994) from which the strategy of cognitive reappraisal originates and to which it ultimately applies. However, there is still no experiment that directly assesses the relationship between cognitive reappraisal’s creativity and its effectiveness in regulating negative emotion.

The aim of this study was to assess this relationship. In Study 1, participants were asked to generate reappraisal with the aim of alleviating and improving the unpleasant feelings that arose from looking at a picture and to then evaluate the creativity and regulatory effectiveness of these reappraisals. We found that it was difficult for the participants to generate truly, highly creative reappraisals by themselves; thus, in Study 2, we showed the participants well-prepared reappraisal materials that varied in their creativity and asked them to evaluate the materials’ regulatory effectiveness and creativity. Both experiments demonstrated a positive correlation between reappraisal’s creativity and effectiveness, thus proving that creativity plays an important role in reappraisal.

## Experiment 1

The aim of this study was to test the hypothesis that creativity plays an important role in cognitive reappraisal’s regulation of unwanted emotions. We proposed that all types of reappraisals, by their very nature, could be viewed as a process of cognitive restructuring and contained, to some extent, the components of creativity. The level of creativity of a given cognitive reappraisal could significantly predict its effectiveness in regulating emotion.

We instructed a group of participants on how to generate cognitive reappraisal in response to unpleasant pictures and then asked them to do so by themselves. The participants were then required to evaluate the effectiveness and creativity of their reappraisals. The creativity of these reappraisals was also evaluated by another group of people who had expertise in emotional regulation strategies, including cognitive reappraisal. We predicted that a cognitive reappraisal’s effectiveness in regulating emotion would be significantly correlated with its creativity.

### Materials and Methods

#### Participants

A total of 30 Chinese participants (16 women) with a mean age of 22.2 years (*SD* = 1.6, range = 19–25) took part in this study. They were all undergraduate or graduate students at Capital Normal University. The participants were paid 40 RMBs, and written consent was obtained prior to the experiment. This study was approved by the Ethical Committee of Capital Norman University.

#### Procedure

The participants were tested individually and viewed in 25 negative pictures twice each. The pictures were selected from the International Affective Picture System (IAPS) (Lang et al., 2008) and covered a variety of negative stimuli and situations (e.g., threat/attack scenes, horrible animals, disgusting things, etc.). These stimuli were selected to have a mildly negative valence, with an average score of 2.56 (*SD* = 0.52, range = 1.51–3.55; 9-point scale), and to be arousing, with an average score of 5.43 (*SD* = 0.85, range = 3.93–7.35; 9-point scale).

**FIGURE 1:**
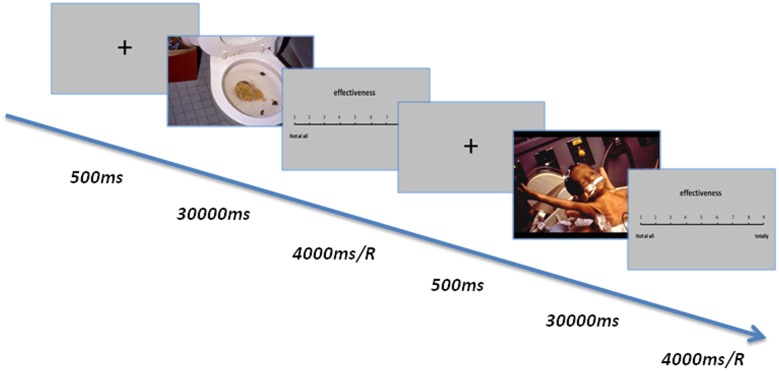
The procedure of experiment 1. The participants were asked to view negative pictures and describe their reappraisals aloud during a 30,000 ms presentation; then, a 9-point scale for effectiveness appeared, and participants were asked to rate the degree of effectiveness in regulating their negative emotion associated with the unpleasant pictures (1 = not at all, 9 = completely). Effectiveness was defined as “the degree or extent to which the reappraisal could improve (which means to decrease the negative emotion or even promote the positive emotion) your feelings toward the picture.”

Participants were asked to view all 25 negative pictures one by one and to describe their reappraisals aloud for each picture during its presentation (30,000 ms) on a screen with the goal of alleviating and improving the unpleasant feelings that arose from looking at the pictures (**Figure 1**). After generating the reappraisal solution, participants were asked to press the button “1,” and the evaluated picture would be presented on the screen. If participants could not generate the reappraisal solution in 30,000 ms, the next picture was presented, and participants were asked to answer the next reappraisal solution immediately. This procedure has typically been used to study cognitive reappraisal (Ochsner et al., 2004; Goldin et al., 2008; Kanske and Kotz, 2011). Immediately after pressing the button, a 9-point scale for effectiveness appeared, based on which participants were to rate the degree of effectiveness in regulating their negative emotion associated with the unpleasant pictures (1 = not at all, 9 = totally). Effectiveness was defined as “the degree or extent to which the reappraisal could improve (which means to decrease the negative emotion or even promote the positive emotion) your feelings toward the picture.” Before the formal procedure of cognitive reappraisal, the participants were instructed and trained on how to perform reappraisals of the unpleasant pictures, which also followed the standard procedure reported in similar studies (Ochsner et al., 2004; Goldin et al., 2008; Kanske and Kotz, 2011). The participants also received 6 training trials prior to the formal experimental procedure to familiarize them with the procedure and to practice the reappraisal strategies. The oral reappraisals were recorded and transcribed for further analysis.

After the participants had generated their cognitive reappraisals for all 25 pictures, they were required to view the same 25 pictures again and to write down their answers to the following 2 questions: (1) What was the reappraisal you made for the picture the last time that you saw it (the memory recall task)? (2) Do you think your reappraisal is a creative one (the self-rating of creativeness)? The participants rated the degree of creativity using a 9-point scale (1 = not at all, 9 = completely). We defined creativity as “difficult to obtain from other people.” Each picture was presented in random order in the first presentation, and the second time, the picture was presented in the same order as the first time for each individual. After the experiment, they were showed a comedy for 10 min with the aim of regulating the uncomfortable feelings that arose from looking at the negative pictures. None of our participants reported feeling uncomfortable during or after the experiment.

The transcribed text of the participants’ cognitive reappraisals and the recalled reappraisals were then rated by 8 graduate students (experts) who majored in related psychological fields (such as psychological counseling) and had knowledge and experiences associated with cognitive reappraisal and emotional regulation on 2 dimensions: (1) the degree of creativity (on a 9-point scale ranging from 1 = not at all to 9 = completely); and (2) the degree of consistency between a given participant’s initial reappraisal of a given picture and their subsequent memories of it (also performed on a 9-point scale). Before making their evaluation, these graduate students were instructed to reach a full understanding of the meaning of “creativity” and “consistency.” Through these evaluation procedures, we wanted to learn (1) how well the participants could successfully recall the initial cognitive reappraisal they had made for the given picture; and (2) the consistency between self-rated creativity and other-rated creativity.

### Results and Discussion

A significant trial-by-trial positive correlation was observed between the participants’ self-rating on the effectiveness of the reappraisal and their self-rating on its creativity (*r* = 0.44, *p* < 0.001, *N* = 748; *M*_self-rating of creativeness_ = 5.41, *SD* = 2.18*; M*_self-rating of effectiveness_ = 6.72, *SD* = 1.91). This result demonstrated that the creative factor might play an important role in a given cognitive reappraisal’s regulating effect on the unwanted emotion. The degree of consistency between the participants’ initial reappraisal and their later memories of it was 7.69 (*SD* = 3.82) on the 9-point scale. This finding implies that the participants’ memories of their initial reappraisal were very reliable. There was a significant, but lower, trial-by-trial positive correlation between the experts’ ratings of creativity and the participants’ self-ratings of creativity (*r* = 0.16, *p* < 0.001, *N* = 748). However, the experts’ ratings of creativity were significantly lower than the self-ratings of creativity [*M*_experts-rating of creativeness_ = *4.68* (*SD* = 0.18) vs. *M*_self-rating of creativeness_ = 5.41 (*SD* = 0.48); *t*(36) = 2.80, *p* < 0.01, Cohen’s *d* = 0.47], and the experts’ ratings of creativity were not correlated with the self-ratings of effectiveness (*r* = 0.07, *p* = 0.07, *N* = 748). These results indicated that (1) the creativity of a given reappraisal could to some extent be objective and could be recognized by both themselves and others and (2) there were fairly large discrepancies between one’s self-rating and the evaluation of others, relative to people who have expertise on the strategies of emotional regulation. The participants might have a tendency to exaggerate the creativity of their own reappraisal. Importantly, we found that the chances of obtaining highly creative reappraisals were very low if we asked people to generate reappraisals on their own. Only 31.9% of the reappraisals were rated as highly creative (≥7 on a 9-point scale) by the participants themselves, and only 0.5% of them were rated as highly creative by the graduate students with relevant expertise. Therefore, to obtain sufficient cases of high-quality creative reappraisal, we should develop a database.

## Experiment 2

Experiment 2 was conducted because we found that it was difficult, if not impossible, for the participants to generate highly creative reappraisals on their own (see Study 1). Therefore, we invited one group participants (75) to generate the creative reappraisal sentences pertaining to unpleasant IAPS pictures and the other group participants (45) to evaluate these materials. It meant that the second group participants were just showed the well-prepared reappraisal sentences that varied in their creativity and asked them to evaluate their regulatory effectiveness and creativity. We collected more than 900 creative reappraisals for the standard IAPS negative pictures from a large group of participants and asked another group of participants to evaluate their creativity, effectiveness and appropriateness.

### Materials and Methods

#### Participants

Two groups of people participated in this study. One group generated the creative reappraisal materials pertaining to unpleasant IAPS pictures, whereas the other evaluated these materials. Seventy-five participants [36 females, with a mean age of 24.5 years (*SD* = 2.31, range = 20–36)], recruited for college students in Beijing, participated in the task of generating creative reappraisal materials. Given that everyone enjoys viewing negative pictures and generating reappraisals of those pictures (indeed, there were some individuals who could not stand viewing the negative pictures or appeared to be averse to generating reappraisals for negative scenes). Therefore, each of the people we recruited were interested in participating in this task and considered themselves highly creative people who could generate creative cognitive reappraisals for these negative pictures. The participants were paid 1–5 RMBs for each reappraisal according to the quality of their work, i.e., the creativity and appropriateness of the reappraisal that they produced for a given picture. Another group of 45 participants [27 females, with a mean age of 24.2 years (*SD* = 1.60, range = 21–28)] took part in the evaluation task. They were paid 150 RMBs for their participation. A written consent was obtained from all participants prior to the task. This study was approved by the Ethical Committee of Capital Normal University.

#### Procedure

**FIGURE 2:**
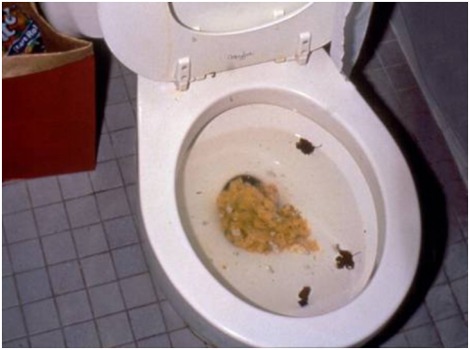
An example of stimuli used in the experiment: Vomitus.

We first selected 75 negative pictures from the International Affective Picture System (IAPS; Lang et al., 2008) based on their normative valence and arousal levels. The mean valence rating was 2.50 (*SD* = 0.45; max: 3.55, min: 1.51), and the mean arousal rating was 5.53 (*SD* = 0.84). We then created a manual that contained detailed instructions on how to approach the 75 selected pictures and sent the manual to the participants through e-mail. In the instructions, we asked the participants to try their best to generate new interpretations (creative reappraisals) that were both novel and appropriate for each unpleasant picture, with the aim of alleviating and improving the unpleasant feelings that arose from looking at the pictures. We also provided examples of highly creative reappraisals for the target pictures in the manual, and the participants were informed that for each reappraisal, they made would be paid 1–2 RMBs but for highly creative reappraisals, they would be paid as much as 4–5 RMBs. Our pilot study found that these procedures could be very helpful for the participants in understanding the requirements of the task and to effectively generate their own creative reappraisals. Only the participants who (a) did not have extremely uncomfortable feelings while viewing the negative pictures and (b) considered themselves able and motivated to generate the creative materials were recruited for the study. The participants were given 2 weeks to think about and write down their reappraisals. After all of the participants returned the creative reappraisal materials, the experimenters, together with students majoring in Chinese language and who were particularly good at wording and phrasing, inspected each item, deleted unqualified descriptions (e.g., those that had no intention to regulate negative emotion, those that exhibited aggressive intentions, or those that were apparently inappropriate for the target picture), combined descriptions with similar meanings and polished all of the satisfactory descriptions. The final database included 946 sentences, the lengths of which were 30–40 Chinese characters. The number of reappraisals/descriptions for each picture ranged from 8 to 23 descriptions. **Figure 2** shows examples of reappraisal descriptions for a given picture. (1) A woman who wanted to have a baby was surprised and found herself to be pregnant when she started vomiting. (2) After a Halloween party, a mother poured leftover pumpkin into the toilet. (3) A girl deliberately poured leftovers into the toilet and had the opportunity to call the boy she liked to help her fix the problem.

These creative reappraisal descriptions were then evaluated by the other 45 participants on 3 dimensions: (1) creativity (the extent to which one feels that these are novel and unexpected); (2) appropriateness (the extent to which the description is appropriate for or fits with the scene depicted in the picture); and (3) effectiveness (the extent to which the description can make one’s feelings and emotion in looking at the picture better) using a 9-point scale ranging from 1 (not at all) to 9 (completely). We predicted that both creativity and appropriateness were significant predictors of effectiveness in regulating negative emotion.

The participants were first instructed to reach a full understanding of the meaning of “creativity,” “appropriateness” and “effectiveness” before beginning the formal procedure. They were then required to input their evaluations of the descriptions, which were presented together with the target pictures one by one. The entire evaluation process was divided into 3 sessions, and each session included 25 pictures and the corresponding descriptions (each session took approximately 2–3 h). All reappraisal descriptions for a given target picture were presented at the same time when the target picture was shown on the screen. This feature was designed for two reasons: (1) If only one part of a reappraisal description for a given target picture was presented, it would take too long to evaluate all pictures, as each target picture would be presented 8–23 times and 25 target pictures would be presented a total of 948 times; (2) participants could directly compare different reappraisal descriptions to which they were exposed at the same time. To avoid confusion and interactions, the evaluations of creativity, appropriateness, and effectiveness were not conducted in the same trial but were evaluated in separate sessions, and the sequence of these three types of evaluations was pseudo-randomly assigned and counter-balanced across participants. After the experiment, they were shown a comedy for 10 min with the aim of regulating the uncomfortable feelings that arose from looking the negative pictures. None of our participants reported feeling uncomfortable during or after the experiment. On the contrary, some of our participants even expressed their willingness to take part in our study again because they felt they were greatly inspired by the examples of highly creative reappraisals and “learn something” from this study.

### Results and Discussion

The average creativity rating of all participants for each piece of creative description ranged from 2.95 to 7.80 (*M* = 5.59, *SD* = 0.86). The effectiveness rating of all parts of reappraisals ranged from 2.48 to 7.33 (*M* = 5.69, *SD* = 0.61), and the appropriateness rating of those parts ranged from 4.05 to 7.68 (*M* = 6.21, *SD* = 0.58). For all creative reappraisals, there was a significant positive correlation (*r* = 0.56, *p* < 0.001) between creativity and effectiveness, which was consistent with the results obtained in part 1. We also found a significant negative correlation (*r* = -0.58, *p* < 0.001) between appropriateness and effectiveness and a significant negative correlation (*r* = -0.60, *p* < 0.001) between creativity and appropriateness, which shows that an increase in creativity was inevitably accompanied by a sacrifice of appropriateness.

Stepwise regression demonstrated that creativity and appropriateness could together explain 44% of the total variance of effectiveness [*F*(2,943) = 370.30, *p* < 0.001), β_creativeness_= 0.83 (*t* = 27.15, *p* < 0.001), β_appropriateness_ = 0.45 (*t* = 14.81, *p* < 0.001)], which shows that both creativity and appropriateness were significant predictors of the regulating effects of the reappraisal and that creativity was the most dominant predictor.

## Discussion

In this study, we assessed the relationship between creativity and cognitive reappraisal by investigating how the regulatory effectiveness of a given reappraisal could be related to its creativity. In Study 1, participants were asked to generate cognitive reappraisals of negative IAPS pictures, and we found that there was a significant positive correlation between the effectiveness of the cognitive reappraisal and its creativity rating, thus proving the role of creativity in cognitive reappraisal’s regulatory effectiveness. However, given that the participants could rarely generate high-quality creative reappraisals by themselves, we prepared, in advance, novel and appropriate reappraisal materials that varied in their creativity and presented these well-prepared materials to the participants together with negative IAPS pictures. Our result again proved the relationship between reappraisal’s creativity and its effectiveness in regulating negative emotion in an emotional regulatory procedure involving passively reading (rather than positively generating) the high-quality creative reappraisals. Both studies demonstrated the critical role of creativity in cognitive reappraisals.

As previously noted, there were already two categories of evidence that indicated the relationship between cognitive reappraisal and creativity: (a) the perspective of individual differences, which indicated that one’s ability to generate different cognitive reappraisals could be predicted by one’s score on openness to experience, a feature that is believed to be strongly related to creativity, as well as one’s performance in divergent thinking (Weber et al., 2014); and (b) the generation process perspective, which indicated that the generation of cognitive reappraisal could be accompanied by a brain activation pattern (and, thus, psychological process) similar to that observed in verbal creative ideation (Fink et al., 2016). However, neither of these perspectives covers regulatory effectiveness, which is undoubtedly the core concern of the cognitive reappraisal strategy. The present study provided the appropriate chain of evidence by proving that a given cognitive reappraisal’s effectiveness in regulating negative emotion could be predicted by its perceived creativity. Our results, together with those obtained from the perspective of individual differences and the mental generation process, proved the essential association between creativity and cognitive reappraisal from a relatively comprehensive person-process-effectiveness perspective.

Accordingly, we proposed a “representational change account” for cognitive reappraisal specific to a given unpleasant situation (such as viewing a negative IAPS picture). Ohlsson and Knoblich established representational change theory for creative insight problem solving, which suggests that problem solvers initially have a low probability of success because they retrieve inappropriate knowledge and set unnecessary constraints on the problem and that only when they break these inappropriate mental sets and establish a new but suitable representation of the problem can they have success in a seemingly unsolvable insight problem (Ohlsson, 1984, 1992; Knoblich et al., 1999, 2001; Luo et al., 2006; Wu et al., 2009, 2013; Huang et al., 2015; Tang et al., 2016). It is reasonable for us to propose that similar processes can also occur in cognitive reappraisal’s regulation of emotion: when encountering an apparently unpleasant stimulus, such as a picture of vomitus, people’s initial feeling or intuitive emotional response can be disgust; only when they have restructured the situation and given the scene a new meaning (for instance, reinterpreting the vomitus as a sign of pregnancy for a woman who wants to have a baby) can these negative feelings be altered. The application of representational change theory from creative insight problem solving to cognitive reappraisal includes three major points: (1) the individual’s initial mental representations of the situation can lead to negative emotional arousal and cognitive consequences; (2) these negative mental representations function as a type of “mental set” or stubborn cognitive-emotional response bias that is difficult to efficiently change through one’s ordinary mode of rational thinking or stepwise reasoning; and (3) an individual must have insightful creative ideas that are particularly appropriate for reinterpreting the situation in a new manner that can lead to apparently less negative cognitive-emotional consequences. That is, only when an individual generates a novel and appropriate interpretation of the scene that he/she has perceived as negative can he/she efficiently alter the initial negative tendency. The representational change account of cognitive reappraisal emphasizes the role of creativity and insightfulness in this emotional regulation strategy.

A potential difficulty in applying the creative principle of cognitive reappraisal to practice is that it will be difficult, if not impossible, for individuals to generate reappraisals that are truly creative. Study 1 clearly demonstrated that only one-third of the self-generated reappraisals were rated as highly creative by the participants who generated the reappraisals themselves, and according to the rating of the graduate students with relevant expertise, only 0.5% of these reappraisals were rated highly “creative” (≥7). From an evolutionary perspective, negative stimuli are closely related to survival, and it can be easier for such stimuli to automatically and preferentially induce negative emotions. Negative emotions make people narrow their focus of attention (Frolli et al., 2016), focusing all their mental energy on the negative emotion. At that moment, however, spontaneously generating a highly creative cognitive reappraisal requires one not only to break from the constraints of dominant negative emotions but also to develop a creative interpretation that is both novel and appropriate. This process requires a considerable amount of initiative and consumes more psychological resources. Under these circumstances, individuals typically tend to adopt a fixed mode of thinking to generate their reappraisal, such as “this is not true,” “things will be better” and “this picture content has nothing to do with myself,” because this strategy can help them save their depleted cognitive resources. The results of this ordinary thinking strategy, however, can eventually lead to a mediocre reappraisal and can cause it to lose its insightful nature and remarkable efficiency.

As demonstrated in Study 2, one method of solving this “generation difficulty” is to present high-quality creative cognitive reappraisal materials that are elaborately prepared by others. The advantage of this approach is that it does not require the individual to generate the creative reappraisal him/herself. Moreover, because the reappraisal made by others can be more unexpected and novel to individuals than the reappraisal made by their own self, it has a greater chance of helping individuals establish a new, favorable mental representation. The restriction of this approach, however, is that it requires a relatively clear definition of the problem situation and a universally recognized creative reinterpretation; meeting these two requirements can be difficult under real psychological conditions. For example, it may not be easy for an individual who is experiencing psychological problems to be clearly aware of what exactly his/her problems are, develop a creative reappraisal of them. Yu et al. (2015) attempted to use low- and high-restructuring (creative) solutions for coping with real psychological distress (“problem”). By pooling the psychological issues that college students frequently encounter in everyday lives, Yu et al. (2015) identified a list of typical problems (for example, “*I feel extremely frustrated because of beginning a career I dislike*”) and created corresponding reappraisal-like counseling dialogs for these problems. The authors found that the relatively high-restructuring (creative) dialogs (for example, “*Success in life is not holding good cards but playing bad cards well*” for the above mentioned problem) can be more “insightful” (which refers to the extent to which there is a cognitive “click” or new enlightenment that would be helpful in understanding life’s puzzles) than the non-creative, ordinary dialogs (“*Success mainly depends on effort; it is important to do a good job now*”). However, these restructuring dialogs are, by nature, metaphorical expressions of vaguely defined real-life issues; thus, the creative components of these dialogs still cannot be clearly identified, as in the creative reappraisals that we used in this study, because the IAPS pictures typically depict relatively simple and clear problem situations for which creative solutions can reasonably be formulated. How we can obtain psychotherapeutic problems and dialogs whose creativity or, more specifically, whose key components of representational change can be more precisely defined remains to be investigated?

Another issue that may have theoretical implications is the appropriateness rating. Although appropriateness and novelty have been widely recognized as the essential features of creativity (Barron, 1955; Runco and Jaeger, 2012; Huang et al., 2015), the present study indicated an inverse relationship between the creativity and appropriateness ratings. Because the scene that an IAPS picture depicts and its implications are typically clear and unambiguous, the most appropriate manner in which to interpret the picture is to objectively describe it. Therefore, any reappraisal efforts that alter the original meaning of the picture can lead to a decrease in appropriateness. Thus, an increase in creativity may inevitably lead to a decrease in appropriateness. Fortunately, the present study found that creative reappraisals could still regulate emotion with high efficiency if their appropriateness reached a certain level. Thus, an individual may have a certain degree of tolerance in accepting reappraisals with imperfect appropriateness. It could be interesting to investigate why and the extent to which individuals can accept a reappraisal that is less perfect in its appropriateness and the role that other factors, such as internal motivation and the reappraisal’s perceived creativity, play in this procedural tolerance.

Thus, in this study, we proved our assumption that creativeness is an important factor for a cognitive reappraisal to regulate negative emotion by illustrating the significant correlation between the self- or other-rated creativity and the effectiveness of cognitive reappraisal. Nevertheless, some issues must be more specifically addressed in future research. First, our study only investigated the role of the positive regulative intention of creativity in emotional regulation. However, highly creative reappraisal includes not only positive regulative intention but also negative regulative intention solutions. For example, for the picture of vomitus, the solution of the pregnancy of a woman who wants to have a baby is a positive regulative intention solution; however, if we reappraise the picture as vomitus after knowing that she ate something that was left over after being eaten by a dog, although both are unexpected or creative reappraisals, the efficiency of emotional regulation may be different. The emotional regulative intention may be an important factor and must be investigated in future studies. Second, the materials that we have established could be adapted to examine the brain mechanism underlying creative cognitive reappraisal, which is important and valuable for real-life emotional regulation. Third, in our study, all stimuli were selected to have a mildly negative picture, and extremely negative scenes (such as died bodies) were excluded. The applicability of the creative reappraisal strategy should be considered in conjunction with the severity of the negative situation. In our future work, we want to determine whether extremely negative scenes could be regulated using the cognitive reappraisal as mildly negative pictures. Finally, we have considered the contribution of reappraisal’s creativeness and appropriateness to its efficiency; however, we should also keep in mind that other situational and emotional factors could alter the regulatory effects of reappraisal, such as stress. The study performed by Raio et al. (2013) suggested that although the cognitive reappraisal could reduce the subjective angry feeling evoked, this regulatory effect was no longer observed when participants were in a stress state. For creative cognitive reappraisal, we want to determine whether there is a similar regulatory effect in a stress state.

## Author Contributions

TG and JL designed and supervised the study. TG collected the data. TT analyzed the data. BS, JL, and XW wrote the manuscript.

## Conflict of Interest Statement

The authors declare that the research was conducted in the absence of any commercial or financial relationships that could be construed as a potential conflict of interest.
